# Alveolar Abscess

**Published:** 1849-07

**Authors:** J. Taylor


### ALVEOLAR ABSCESS.

BY J. TAYLOR, M. D., D. D. S.

Alveolar Abscess—Gum-Bile: This disease is "a collection of pus in a sac formed in the socket of a tooth at the extremity of a root, which generally escapes through the gum."—*Harris' Dictionary of Dental Science.*

The cause of this disease is perhaps universally admitted to be an inflamation in the lining or investing membrane of the tooth; or in other words, the Periosteum; and is generally the sequence of that inveterate kind of tooth ache, which baffles all the ordinary applications for relief, and has universally killed, and will forever kill the reputation of all the specifics and panaceas for this horrible disease. A physician might as well expect to cure an abscess on the lungs, by the introduction of a few drops of some odontalgic remedy, on a pledget of lint, into the nostril of his patient; as for the dentist to expect to cure this disease by the use of the same drops applied to the teeth. We have never known an abscess to form (of this kind) until after the nerve of the tooth had been destroyed. The inflamation which destroys the nerve itself, most usually somewhat abates before that sets in, which results in the formation of an abscess at the point of the root. Sometimes, often indeed, it entirely subsides, leaving an imperceptible chronic form of disease, where the nerve has penetrated the investing membrane. This is at some future time renewed by some exciting cause; such as cold, a sudden jar on the teeth, irritation caused by dental operations, &c. It matters not, however, what may be the cause which brings on the attack, the result will generally be the same; the inflamation will pass through its different stages, and end in suppuration. Let us look a moment at these different stages; the symptoms which characterize the same, and the treatment which they suggest. Every dentist and physician should know from the symptoms of each case of odontalgia called on to treat, whether it is of such a nature as will, in all probability, terminate in Alveolar abscess. In speaking of this disease we must necessarily do so in connection with what is generally called *tooth ache*, for it is still often regarded as the same, and spoken of as such. Indeed, all pain about the jaws until of late, was regarded as *tooth ache*—now the more fashionable term neuralgia is generally applied. This is owing perhaps to the fact that odontalgia often runs into alveolar abscess, and the symptoms attending the formation of this disease, are more generally described by our dental authors in connection with the former disease, than with the latter. Our object is not to go into the general character of inflamation; but merely give as we think, such description of the symptoms, as shall enable us to form a correct diagnosis of the disease. Mr. Robinson, in speaking of odontalgia, gives the following classification—1st, inflamation of the dental nerve; 2d, fungus of the pulp; 3d, confinement of matter in the internal cavity of the tooth; 4th, disease of the periosteum; 5th, sympathy.

In inflamation of the nerve or pulp, the pain is sharp, lancinating and throbbing, and is not increased by pressure on the part. In tooth ache, originating from the confinement of pus in the dental cavity, "pain is felt only at first when hot or cold liquids pass over the tooth;" after this a more constant, steady pain sets in. He then goes on to say, "that when a tooth first grows painful in this way, that it is about to suppurate; when it appears longer than the rest, loose and exceedingly sore, the nerve has suppurated, and the pus is already oozing from the end of the fang, where the vessels and nerve enter. When the cheek begins to swell, it is a sign that the matter is spreading between the alveolus and its lining membrane; which occasions the throbbing pain felt during the formation of alveolar abscess.

Dr. Harris remarks that "while the inflamation is confined to the lining membrane and dental pulp, although the pain is generally very severe, it is not increased by pressure upon the tooth; nor does the affected organ appear to be raised in the least from the socket. But after the inflamation has extended to the alveolar dental membrane, a slight protrusion of the tooth as before stated, takes place. The pain continues, and in some cases is followed by considerable constitutional disturbance; such as dryness of the skin, head ache, fullness and increased rapidity of the pulse, flushed cheeks, &c., &c. At length the pulp suppurates, and an abscess forms at the extremity of the root, which is sooner or later discharged through the alveolus and gums; when the patient is relieved."

We do not think it essential in what we have to say in relation to alveolar abscess, to go into the classification of Mr. Robinson; for an inflamation existing in a fungous growth of the pulp, would exhibit about the same variety of symptoms as that of the nerve itself. We would, however, here remark, that an inflamation in this case, which would destroy the nerve, would also destroy the fungous growth of the pulp. It is but seldom, however, that these fungous growths are very painful, except when they are pressed upon by hard particles of food, or something of that kind introduced into the mouth. The pain consequent upon the confinement of matter in the dental canal, unless removed by an opening in the tooth, must result in alveolar abscess; and the character of the pain is perhaps always the same, except indeed, it may be characterized by the pain felt by the contact of cold or hot substances brought in contact with the tooth. We are not, however, prepared to say that this symptom is at all characteristic of the confinement of matter in the tooth: for we often meet with the same, where such cannot be the case. Teeth, the nerves of which have been destroyed and afterwards filled, often show the first indication of trouble by the application of either hot or cold substances; most generally cold producing pain, while heat abates it, or *vice versa;* yet if the plug is removed, and the nerve cavity thoroughly opened, it does not always afford relief.

The remarks quoted above from Dr. Harris' work—see third edition, page 321, article Tooth ache—would most generally lead the reader to suppose that inflamation in the lining membrane always results in the formation of alveolar abscess, by extending itself to the investing membrane. Such we are satisfied cannot be the Doctor's intention; for in perhaps a large majority of cases, "the inflamation which destroys (as I before remarked) the nerve (and lining membrane) abates before that sets in, which results in the formation of an abscess." This is specially the case where remedies have been used, as is generally the case, for relief of pain. An application of creosote and arsenic for instance, soon kills the nerve and relieves the pain, and in nine cases out of ten, weeks or months will elapse before pain is once more felt. We have now a condition of things favorable for the developing of that disease of which we are treating. What then are the symptoms which will guide us in a correct diagnosis. The first symptom will be a slight, uneasy sensation in the tooth, or rather the alveolus of the affected tooth. This is often described by our patients as a grumbling or growling of the organ. Hours, sometimes days pass on, the tooth becomes a little sore to the touch; yet continued pressure often affords relief. But this pain and soreness increasing, the tooth appears to elongate; this shows that the investing membrane has thickened, so as to raise the tooth in the socket—the tooth gets loose, occlusion of the jaws is painful and difficult—at least where there are antagonizing teeth to the one affected. Redness and slight swelling of the gums around the tooth is now apparent, and that constant, deep throbbing pain, spoken of by Harris and others comes on, showing that matter is being formed at the apex of the root; and as this continues to be deposited, the inflamation extends—the face swells—considerable constitutional derangement often follows, showing itself in a general nervous irritable condition of the system—head ache—full and increased pulse—flushed cheeks—"paroxysms of heat and cold"—hectic fever, &c., &c.; a fistulous opening now forms for the egress of the matter. The inflamation has now run its course, and the swelling and soreness gradually subside. But it must not be supposed that the patient always gets off without far more and serious difficulty. As a general rule, this is all that may be apprehended when the disease is located in the socket of the Incisors, Cuspids and Bicuspides above; but if in the molars, or dens sapientia above, more of the soft parts are implicated; when in the dens sapientia, impeding deglution, and the weight of the matter forcing itself downwards and backwards, is sometimes discharged through the cheeks opposite the inferior molars; if in the molars, the roots may penetrate the antrum, and bring on abscess in that cavity. There is always danger of the fistulous opening through the cheek, when the disease is seated in the socket of any of the under teeth. But by far the most formidable cases of this disease occur when located in the alveolus of the dens sapientia of the inferior maxilla. Here the inflamation often extends to the tonsil glands, to the muscles of the neck, cheek, temples and eyes, impeding deglution, and causing such a rigid condition of the muscles as to prevent opening of the mouth; the matter producing "necrosis and exfoliation" of the alveolar processes, extending forward to the sockets of the molars and Bicuspides, and discharging itself exteriorly at the most depending portion of the jaw; or it takes its course down the neck, and makes an outlet there. These teeth should never be left in the mouth in an aching condition.

Treatment.—When the first symptoms spoken of are apparent, and they can be traced to a dead tooth or root in the mouth, they should be removed; all further difficulty would then be obviated. But this not being the case, and not practicable, a full dose of epsom salts, and a warm bath, the patient kept in bed and well covered; and should the nervous system show much derangement, follow the operation of the salts, with one or more doses of pulv. Doveri, until rest and relief are obtained. Should, however, inflamation not abate, but the tooth become sore and apparently elongated, one or more leeches should be immediately applied, an antiphlogistic regimen kept up, counter-irritation applied to the gum, *not the cheek*, by the application of anodyne liniment, opodeldoc, or the holding of warm water, strongly impregnated with spirits camphor and laudanum in the mouth; and the following treatment for this stage of the disease, recommended by Dr. Harris, will often be found serviceable; "hot salt, moistened with vinegar and laudanum," to be applied externally. We regard, however, this treatment as specially applicable, previous to formation of the sac at the apex of the root, and the deposite of matter.

The following from Dr. Harris' work on the treatment of this disease—see page 435—so well accords with our own views, that we give them in this place. He remarks that the "application of fomentations and emolient poultices to the face, is, perhaps, under hardly any circumstances, advisable; unless when the disease is seated in the alveolus of a front tooth, (we would say in the superior maxilla,) when there is no danger of an external opening; and even then, I do not know that they are productive of any advantage. When the disease is seated in the socket of a molar tooth, external fomentations tend in a greater or less degree, to promote the passage of matter through the cheek." The object of such applications being to arrest inflamation, and prevent the formation of matter, they should never be applied after this has taken place; true, such applications will relax the parts, and afford some temporary relief; but they retard the discharge of matter, and invite it externally. The following, taken from Cooper's Surgical Dictionary, will show the views of one of our most celebrated surgeons on this subject: "How far suppuration can be usefully promoted by medicines or applications is questionable; but attempts are generally made, and for this purpose suppurating cataplasms and plasters, composed of the warm gums, seeds, &c., were formerly much recommended. Mr. Hunter doubted whether such applications had any considerable effect in the way intended; for if they were put on a sore, they would hardly increase the discharge from it; and perhaps even diminish it. However, in many cases in which the parts are indolent, and hardly admit of true inflamation, in consequence of which, a perfect suppuration cannot take place; stimulating the skin brings on a more salutary, and of course, a quicker inflamation; thus the antimonial ointment, and blistering the skin over chronic swellings and abscesses, are sometimes indicated; (Hunter.) We might extend this extract, and show the advantages we might expect from the application of emolient poultices, &c.; yet this would be only applicable when we wished to invite the opening for the matter externally. This we believe is the treatment adopted by most of our physicians in this disease. Now it should be borne in mind that first, the inflamation here existing is not of a chronic character, and second, that the matter should never be encouraged to find its exit through the cheek; blistering the face, therefore, we regard in all cases as entirely unnecessary, and often very injurious; and just so the application of cataplasms and emolient poultices.

When the suppurative process is therefore once established, all these external applications should be dispensed with, merely bandaging the face with a handkerchief, to keep off the cold air which dries the skin, and adds to the pain. Febrile symptoms should be kept under by constitutional treatment; roasted figs may be applied to the gum at that point to where we would invite the matter. This application may really do more good to the mind of the patient, than to the hastening of the egress of the matter; but as soon as we find that that the matter has formed and found its way through the alveolus, so that a puncture with the lancet may reach it, this should be done. A slight elevation of the gum is generally apparent, the most elevated point of which is generally lightly colored by the matter underneath, and unless far back in the mouth, fluctuation is generally perceptible. The opening should always be made with the lancet through the gum, and not through the cheek. We have never seen a case where this would be an exception, except in one or two abscesses formed in the socket of the dens sapientia inferior, and then only when it had been suffered to remain so long that the abscess would have in a short time, burst externally. The foolish prejudice which exists to the extraction of teeth, when the face is thus swollen, and an abscess formed, is without any excuse at all; (except, indeed, the dread of the operation,) for the removal of the tooth is the most direct way of opening the abscess, and the only way, indeed, of getting rid forever of the disease.

When the disease is in the socket of the dens sapientia below, and from the rigid condition of the muscles, the mouth is firmly closed, so that a forcep cannot be introduced, this tooth can be very easily removed by the use of a strong elevator, the *bar* so bent as to enable it to be introduced between this, and the posterior molar tooth; except, indeed, when there is an antagonizing tooth, and this is pressed hard upon it. If this be the case, the mere loosening of this tooth will most generally permit the matter to escape, and in two or three days, the mouth can be so far opened, as to permit the tooth to be entirely removed.

We can scarce conceive of a condition of things where a patient would not be far more able to endure the removal of any ordinary tooth, than to suffer the long continued pain and constitutional derangement attendant upon the formation of alveolar abscess.

